# MET/SMAD3/SNAIL circuit mediated by miR-323a-3p is involved in regulating epithelial–mesenchymal transition progression in bladder cancer

**DOI:** 10.1038/cddis.2017.331

**Published:** 2017-08-24

**Authors:** Jiangfeng Li, Xin Xu, Shuai Meng, Zhen Liang, Xiao Wang, Mingjie Xu, Song Wang, Shiqi Li, Yi Zhu, Bo Xie, Yiwei Lin, Xiangyi Zheng, Ben Liu, Liping Xie

**Affiliations:** 1 Department of Urology, First Affiliated Hospital, School of Medicine, Zhejiang University, Hangzhou, Zhejiang Province, China; 2 Department of Urology, Zhejiang Provincial People’s Hospital, Hangzhou, Zhejiang Province, China; 3 Department of Urology, Tongde Hospital of Zhejiang Province, Hangzhou, Zhejiang Province, China

## Abstract

Bladder cancer (BCa) is the one of the most common cancers with high incidence, occurrence and low 5-year survival rate. Emerging evidence indicates that DLK1-DIO3 genomic region especially the miRNA cluster in this region is involved in several pathologic processes and various cancers, and miR-323a-3p is a member of this miRNA cluster. In this study, we investigate the function and regulatory network of miR-323a-3p in BCa. miR-323a-3p is frequently downregulated in BCa tissues and three cell lines compared with adjacent non-tumorous tissues and bladder normal cell line (SV-HUC-1). Besides, downregulation of miR-323a-3p is significantly associated with poor overall survival rate of BCa. Methylation of DLK1-MEG3 intergenic DMR (IG-DMR) contributes to the reduction of miR-323a-3p. Overexpression of miR-323a-3p significantly inhibits the epithelial–mesenchymal transition (EMT) progression of BCa. Both upregulated MET and SMAD3 are direct targets of miR-323a-3p, and the knockdown of MET and SMAD3 also represses the EMT progression consistently with overexpression of miR-323a-3p. SNAIL is detected in the last targeted confocal protein of both MET and SMAD3 signaling that trigger EMT consequently. Hence, a miR-323a-3p/MET/SMAD3/SNAIL circuit is established to regulate the EMT progression of BCa. And a mutual regulatory mechanism between miR-323a-3p/miR-433/miR-409 and MET also participates in this circuit. In conclusion, our study demonstrates a novel regulatory mechanism of the miR-323a-3p/MET/SMAD3/SNAIL circuit that is involved in the EMT regulation of BCa, which may be a potential therapy target for BCa.

Bladder cancer (BCa) is the ninth most frequently diagnosed cancer in the world currently. According to the data from 1990 to 2012, new BCa diagnoses rose 1.5-fold during 12 years. Although the incidence rates are consistently higher in men than women, stabilizing or declining rates in men but some increasing trends were seen for women in many countries. For the mortality rates, BCa ranks the 13th, and deaths rose 1.3-fold during the 12 years. Similarly, diverging death trends were also observed by sex. Besides, in women patients, it usually occurred that a more advanced disease at presentation and less favorable outcomes after treatment, and deaths of smoking-related BCa are increasing among women in developed countries.^1, 2, 3, 4, 5^ Approximately one-third of BCa patients develop locally advanced and metastatic disease.^6^ Moreover, once BCa becomes metastatic, the 5-year overall survival is a dismal 6%.^7^ Owing to its high incidence and low 5-year survival rate, there is an urgent and meaningful demand of investigating the specific mechanisms and treatment of BCa.

microRNAs (miRNAs) belong to one of noncoding RNAs consisting of 20–23 nucleotides. They are novel gene regulators that target the 3′-UTR of downstream mRNA to accelerate the degradation and/or block the translations of them. Emerging findings of miRNAs have been reported to regulate the cancer progression including BCa. Previously we have identified a series of miRNAs, including miR-26a, miR-101, miR-124-3p, miR-320c, miR-409-3p, miR-490-5p, miR-576-3p, miR-433 and miR-148a that were involved in progression of BCa.^8, 9, 10, 11, 12, 13, 14, 15^

Recently one of the largest miRNA clusters with 53 miRNAs in the forward strand and one (miR-1247) in the reverse strand was found in DLK1-DIO3 genomic region, and many of those miRNAs are differentially expressed in several pathologic processes and various cancers.^16^ Moreover, emerging evidence has confirmed that the silence and low expression of some miRNAs in this cluster are regulated by the methylation of DLK1-MEG3 intergenic DMR (IG-DMR) and MEG3-DMR.^17, 18^ Interestingly, miR-323a-3p and previous identified miR-433 and miR-409 are all transcribed from this cluster. Nevertheless, the mechanisms of miR-323a-3p in regulation of BCa and the relationship with DLK1-DIO3 region are still elusive.

In this study, we demonstrated that downregulated miR-323a-3p due to IG-DMR methylation functioning as a tumor suppressor inhibited the epithelial–mesenchymal transition (EMT) progression by regulating MET/SMAD3/SNAIL circuit. Both MET and SAMD3 targeted by miR-323a-3p were key inducers involved in the progression of EMT, and consequently SNAIL was the last confocal protein to trigger EMT. Thus, a novel regulatory mechanism of miR-323a-3p/MET/SMAD3/SNAIL circuit was established in BCa. Moreover, we also described the mutual regulatory mechanism between miR-323a-3p/miR-433/mir-409 and MET in this circuit.

## Results

### miR-323a-3p is downregulated in BCa

**Figure 1:**
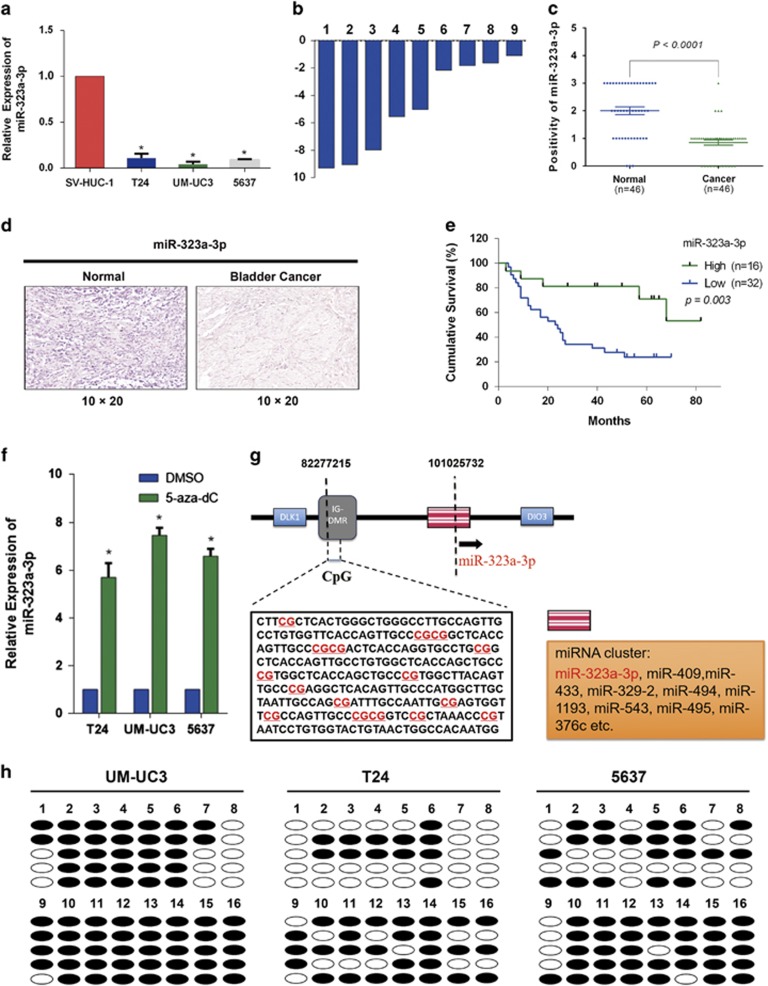
miR-323a-3p is frequently downregulated and modulated by the methylation of IG-DMR. (**a**) The relative expression of miR-323a-3p was significantly decreased in three BCa cell lines (T24, UM-UC3, 5637) compared with the normal bladder cell line (SV-HUC-1). (**b**) The relative expression levels of miR-323a-3p in individual nine pairs of BCa tissues were presented as the fold change of miR-323a-3p referred to the corresponding adjacent normal tissues. (**c**) The CISH results of TMAs indicated that miR-323a-3p was significantly downregulated in BCa tissues than adjacent non-tumor tissues. (**d**) The representative images of CISH of TMAs. (**e**) Downregulation of miR-323a-3p was significantly associated with poor overall survival rate of BCa. (**f**) An obvious upregulation of miR-323a-3p in T24, UM-UC3 and 5637 cell lines after the treatment with demethylation agent 5-aza-CdR. (**g**) The location of miR-323a-3p within the DLK1-DIO3 genomic imprinted miRNA cluster and CpG islands region of IG-DMR analyzed by BSP were all presented. (**h**) Methylation profile of UM-UC-3, T24 and 5637 cell lines. The open and filled circles represent the unmethylated and methylated CpG islands respectively. Five clones from each cell line were analyzed. Error bars represent the S.E. obtained from three independent experiments; *P<0.05

Quantitative real-time PCR (qRT-PCR) was conducted to evaluate the expression level of miR-323a-3p in BCa, and the results revealed the low expression of miR-323a-3p in three BCa cell lines (T24, UM-UC3, and 5637) compared with the normal bladder cell line (SV-HUC-1) (Figure 1a). In addition, nine pairs of clinical BCa tissues and adjacent non-cancerous tissues (the clinical data of the patients are listed in Supplementary Table S1) were also detected by qRT-PCR, and the expression of miR-323a-3p in all BCa tissues was downregulated than adjacent non-cancerous tissues (Figure 1b). Detection of miR-323a-3p expression in BCa tissue microarray (TMAs) with CISH method also demonstrated that miR-323a-3p was significantly downregulated in BCa tissues than adjacent non-tumor tissues (*P*<0.0001, Figures 1c and d). Kaplan–Meier analysis revealed that downregulation of miR-323a-3p was significantly associated with poor overall survival rate of BCa (*P*=0.003, Figure 1e). A further multivariable cox analysis (including sex, age, tumor grade, T stage, lymph node metastasis, miR-323a-3p expression, and SMAD3 expression) also confirmed the same result (*P*=0.047, Supplementary Table S3). All above results indicated the potential role of miR-323a-3p in the carcinogenesis of BCa.

Recently, Leonidas Benetatos *et al.* discovered the DLK1-DIO3 genomic region, which was located on human chromosome 14(14q32). This region not only contained the paternally expressed imprinted genes (DLK1, RTL1, and DIO3), the maternally expressed imprinted genes (MEG3 and MEG8) and antisense RTL1 (asRTL1), but also a miRNA cluster, which was differentially expressed in several pathologic processes and various cancers.^16^ Emerging evidence has indicated that the silence and low expression of some miRNAs in this cluster was regulated by the methylation of IG-DMR and MEG3-DMR, and miR-323a-3p was also involved in this region.^17, 18^ To investigate the mechanism of low expression of miR-323a-3p, we treated the T24, UM-UC3 and 5637 cell lines with 5-aza-2′-deoxycytidine (5-aza-CdR), and redetected the expression of miR-323a-3p. We found a significant upregulation of miR-323a-3p after the treatment (Figure 1f). We further performed the bisulfite-sequencing PCR (BSP), which revealed the high methylation level of IG-DMR in above three BCa cell lines (Figures 1g and h). All the results indicated the high level methylation of IG-DMR contributed to the downregulation of miR-323a-3p in BCa.

### Overexpression of miR-323a-3p inhibits the migration and invasion by regulating EMT progression in BCa

**Figure 2:**
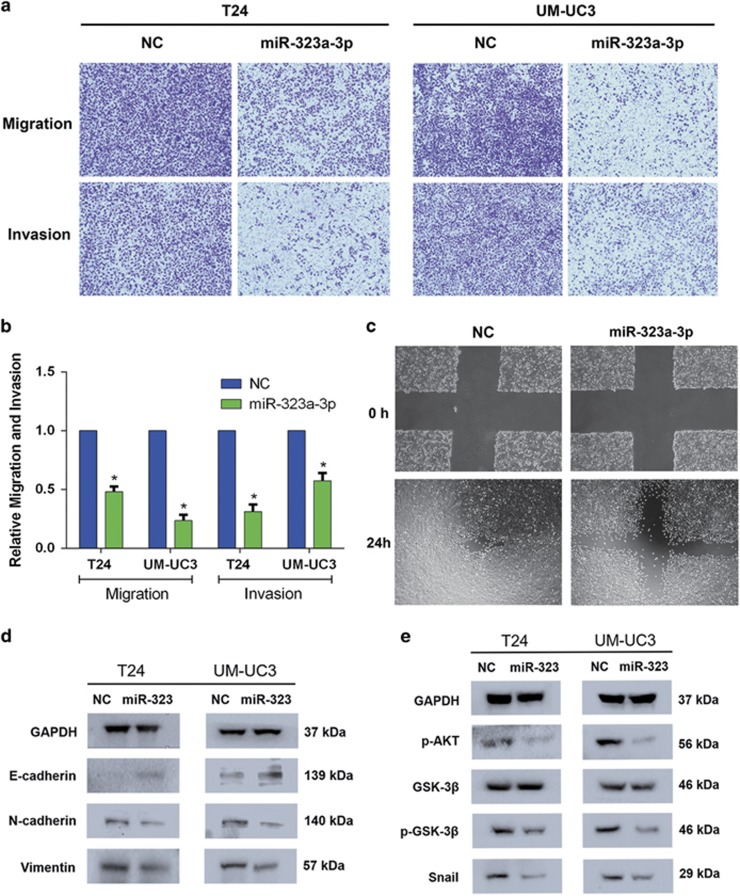
Forced expression of miR-323a-3p inhibits the motility of T24 and UM-UC3 cell lines. (**a**) A trans-well assay revealed an evident inhibition of migration and invasion of T24 and UM-UC3 cell lines transfected with miR-323a-3p (50 nM). (**b**) The representative micrograph of trans-well assay was calculated. (**c**) A wound healing assay showed the consistent inhibition of motility in T24 cell lines. (**d**,**e**) Western blot analysis confirmed that miR-323a-3p (50 nM) inhibited EMT and AKT/GSK-3*β*/SNAIL signaling-related proteins in T24 and UM-UC-3 cell lines. Error bars represent the S.E. obtained from three independent experiments; *P<0.05. Photographs of trans-well assay were obtained under × 10 objective

Overexpression of miR-323a-3p was performed by transfecting miR-323a-3p mimics into T24 and UM-UC3 cell lines, and the transfection efficiency was confirmed by qRT-PCR (Supplementary Figure S1). After a highly efficient transfection, the CCK-8 test, colony formation assay and cell cycle assay were all examined to evaluate the effect of miR-323a-3p on proliferation; however, no significant results were observed (Supplementary Figure S2). But we detected a significant inhibition of cell migration and invasion after the transfection of miR-323a-3p mimics, and the results were tested and confirmed by trans-well and wound healing assay (Figures 2a–c). Previous studies demonstrated that the activation of AKT increases the nuclear expression and transcriptional activity of SNAIL via the inhibitory phosphorylation of GSK-3*β*, thereby triggering cell migration and EMT, and this signal was also detected in BCa.^19, 20^ Western blot was performed and the results suggested that miR-323a-3p inhibited the migration and invasion similarly via blocking AKT/GSK-3*β*/SNAIL signal and EMT progression (Figures 2d and e). All the results revealed that miR-323a-3p functioning as a tumor suppressor inhibited BCa migration and invasion by regulating EMT progression.

### Both MET and SMAD3 are direct targets of miR-323a-3p

**Figure 3:**
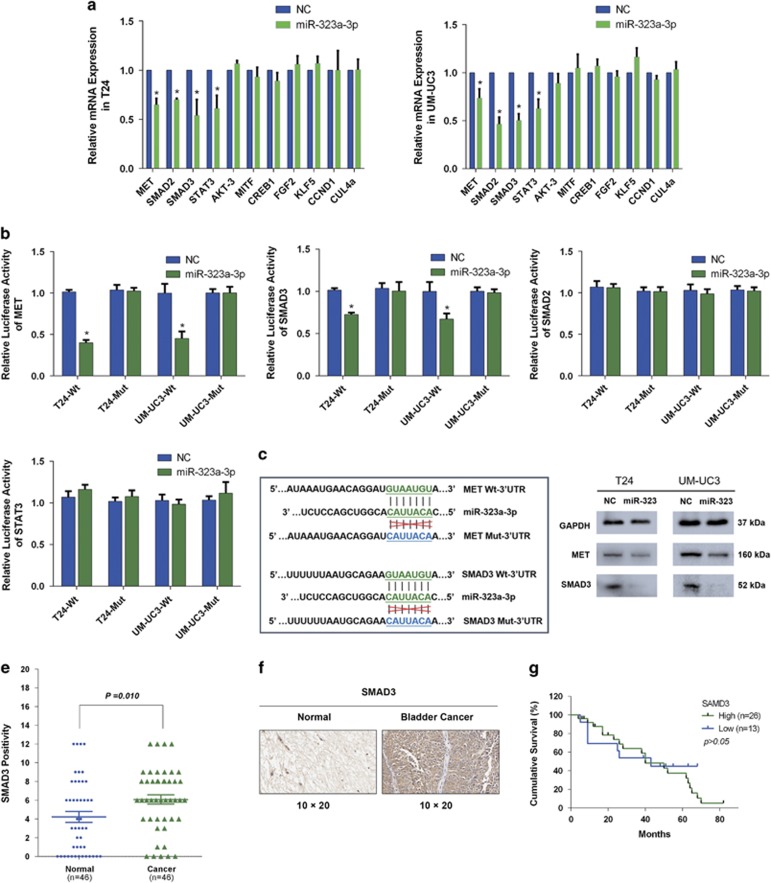
Both upregulated MET and SMAD3 in BCa are direct targets of miR-323a-3p. (**a**) A qRT-PCR assay detected the significant downregulation of MET, SMAD2, SMAD3 and STAT3 in T24 and UM-UC3 cell lines transfected with miR-323a-3p mimics. (**b**) A dual-luciferase reporter assay indicated miR-323a-3p significantly suppressed the luciferase activity of vectors that carried 3′-UTRs of MET and SMAD3 but not SMAD2 and STAT3. (**c**) The targeting sites of miR-323a-3p in the 3′-UTRs of MET and SMAD3 were mutated as represented. (**d**) Western blot analysis verified that miR-323a-3p repressed the expression of MET and SMAD3. (**e**) SMAD3 was significantly upregulated in BCa tissues than adjacent non-tumor tissues. (**f**) Representative images of IHC staining of SMAD3 in TMAs. (**g**) Kaplan–Meier survival analysis showed that the level of SMAD3 was not associated with overall survival rate of BCa. Error bars represent the S.E. obtained from three independent experiments; *P<0.05

Targetscan (http://www.targetscan.org/) and mirdatabase (http://www.mirdb.org) online databases were utilized to predict and identify the potential targets of miR-323a-3p. After primary screening, we selected some candidate targets to confirm. Subsequently, we detected the mRNA level of the predicted targets by qRT-PCR after the treatment with miR-323a-3p mimics. The results revealed only four candidate targets (MET, SMAD2, SMAD3, STAT3) that were downregulated at mRNA level in both T24 and UM-UC3 cell lines (Figure 3a).

We next performed luciferase reporter assays to confirm the direct interaction between miR-323a-3p and the candidate targets (MET, SMAD2, SMAD3, STAT3). We detected that the relative luciferase activities of MET and SMAD3 were decreased when miR-323a-3p was overexpressed in T24 and UM-UC3 cell lines, but other candidate targets had no same results. As expected, the luciferase activity of the mutated vectors was unaffected by the transfection of miR-323a-3p mimic (Figure 3b). The targeted and mutated sequences of miR-323a-3p were shown as an image (Figure 3c). Western blot analysis showed the protein levels of MET and SMAD3 were also reduced (Figure 3d).

To further confirm the expression of both targets and correlation with miR-323a-3p expression in BCa tissues, immuno-histochemical (IHC) staining of both targets and CISH of miR-323a-3p were performed. Previously, Xu *et al.* has reported that IHC staining of MET was upregulated in BCa tissues than adjacent non-tumor tissues but had no significant association with the overall survival rate of BCa.^20^ Based on above data, we made a further correlation analysis. Interestingly, the negative correlation of miR-323a-3p and MET was found by analyzing the data of CISH results of miR-323a-3p and the IHC results of MET in TMAs (*r*^2^=0.09905, *P*=0.031, Supplementary Figure S3A). Moreover, the IHC staining of SMAD3 indicated that SMAD3 was also upregulated in BCa tissues than adjacent non-tumor tissues (*P*=0.010, Figures 3e and f), and was negatively correlated with miR-323a-3p (*r*^2^=0.09142, *P*=0.0411, Supplementary Figure S3B). Unfortunately, Kaplan–Meier survival curves indicated that the level of SMAD3 was not significantly associated with overall survival rate of BCa (Figure 3g). The levels of MET and SMAD3 had a minor positive correlation tendency, but no statistical significance (*r*^2^=0.05229, *P*>0.05, Supplementary Figure S3D).

Collectively, all these data indicated that the high level of MET and SMAD3 as oncogenes are involved in carcinogenesis of BCa, and both are direct targets of miR-323a-3p.

### Knockdown of MET inhibits EMT and both inhibition of miR-323a-3p and overexpressed MET partially rescue downregulated MET-induced suppression of EMT in BCa

**Figure 4:**
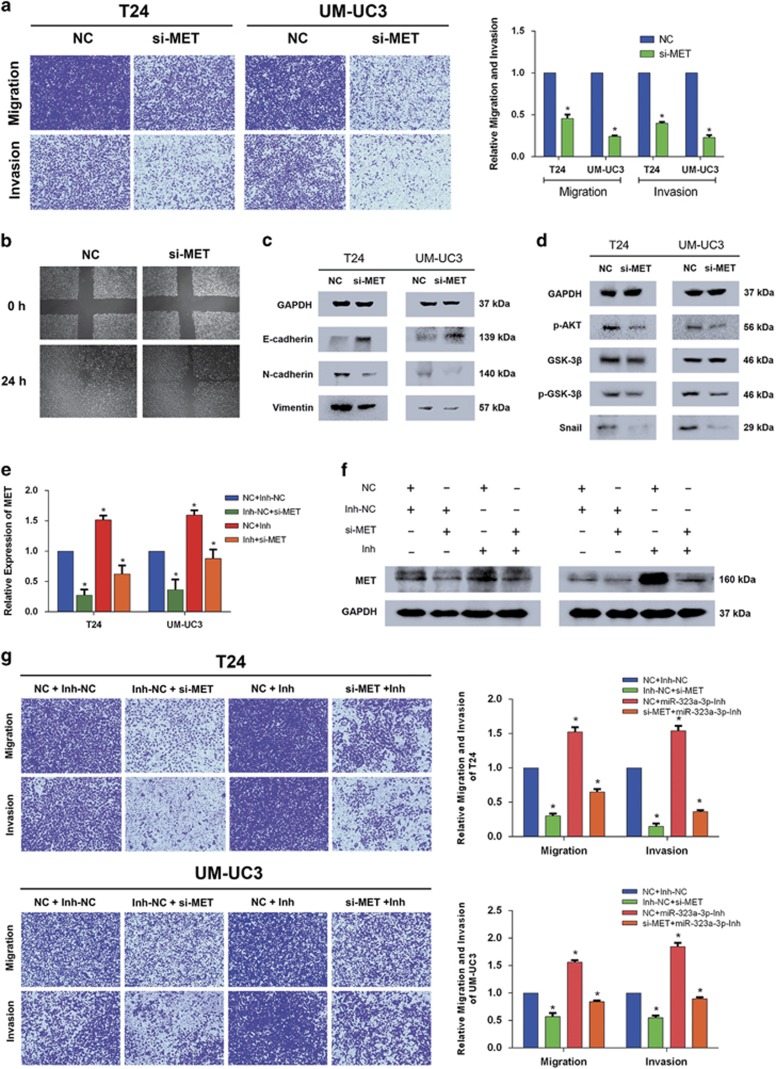
The knockdown of MET impaired the motility of T24 and UM-UC3 cell lines and inhibition of miR-323a-3p expression partially rescues si-MET induced suppression of cell motility. (**a**) A trans-well assay suggested the significant reduction of motility, and the relative migration and invasion rate was calculated. (**b**) A wound healing assay revealed the consistent repression of mobility of T24 cell line. (**c** and **d**) Western blot analysis confirmed that si-MET inhibited the EMT by regulating AKT/GSK-3*β*/SNAIL signal. (**e**) q-RT-PCR revealed that co-transfection of miR-323a-3p-Inh and si-MET attenuated the expression repressed by si-MET at the mRNA in T24 and UM-UC3 cell lines. (**f**) Western blot assay shows the co-transfection of miR-323a-3p-Inh and si-MET attenuated the expression repressed by si-MET at protein level in T24 and UM-UC3 cell lines. (**g**) Trans-well assay consistently indicated that co-transfection of miR-323a-3p and si-MET abrogated the motility of T24 and UM-UC3 cell lines. And the relative migration and invasion rates were calculated. Error bars represent the S.E. obtained from three independent experiments; *P<0.05. Photographs of trans-well assay were obtained under × 10 objective

To confirm the precise effect of MET in BCa, we used siRNAs to knock down MET, and a significant inhibition of migration and invasion was observed by trans-well and wound healing assays (Figures 4a and b). Three different and effective siRNAs were designed to knock down MET to avoid off-target effects, and the effect of repression was demonstrated at both the mRNA and protein levels (Supplementary Figures S4A and B). In addition, we confirmed that MET activated the AKT/GSK-3*β*/SNAIL signal to induce EMT and migration (Figures 4c and d). All results were consistent with the study reported by Xu *et al.*^20^

To further elucidate the direct interaction between miR-323a-3p and MET, we performed two rescue experiments. To begin with, we tested whether the inhibition of miR-323a-3p could abrogate the inhibition of EMT and migration induced by si-MET. q-RT-PCR results indicated that further downregulation of miR-323a-3p induced by miR-323a-3p-Inh (miR-323a-3p-Inh/Inh) significantly upregulated the expression of MET, and co-transfection of miR-323a-3p-Inh and si-MET abrogated the expression of MET knocked down by si-MET (Figure 4e). Moreover, western blot assay consistently confirmed the same results at the protein level (Figure 4f). In addition, the migration and invasion detected by trans-well and wound healing assays showed a partial abrogation induced by miR-323a-3p-Inh (Figure 4g and Supplementary Figure S4C). Secondly, another experiment with overexpressed plasmid (p-MET) method also confirmed the same results. Trans-well assay revealed that only overexpression of MET significantly induced the migration and invasion, and co-transfection with overexpressed plasmid (p-MET) and miR-323a-3p reversed the migration and invasion inhibited by miR-323a-3p in T24 and UM-UC3 cell lines (Supplementary Figures S5A and B). The changes of protein level were presented consistently with the phenotype(Supplementary Figure S5 C).

To sum up, all of the evidence suggested that MET as a key direct target of miR-323a-3p was involved in the migration and metastasis of BCa.

### Knockdown of SMAD3 inhibits EMT and both inhibition of miR-323a-3p and overexpressed SMAD3 partially rescue the downregulated SMAD3-induced suppression of EMT in BCa

**Figure 5:**
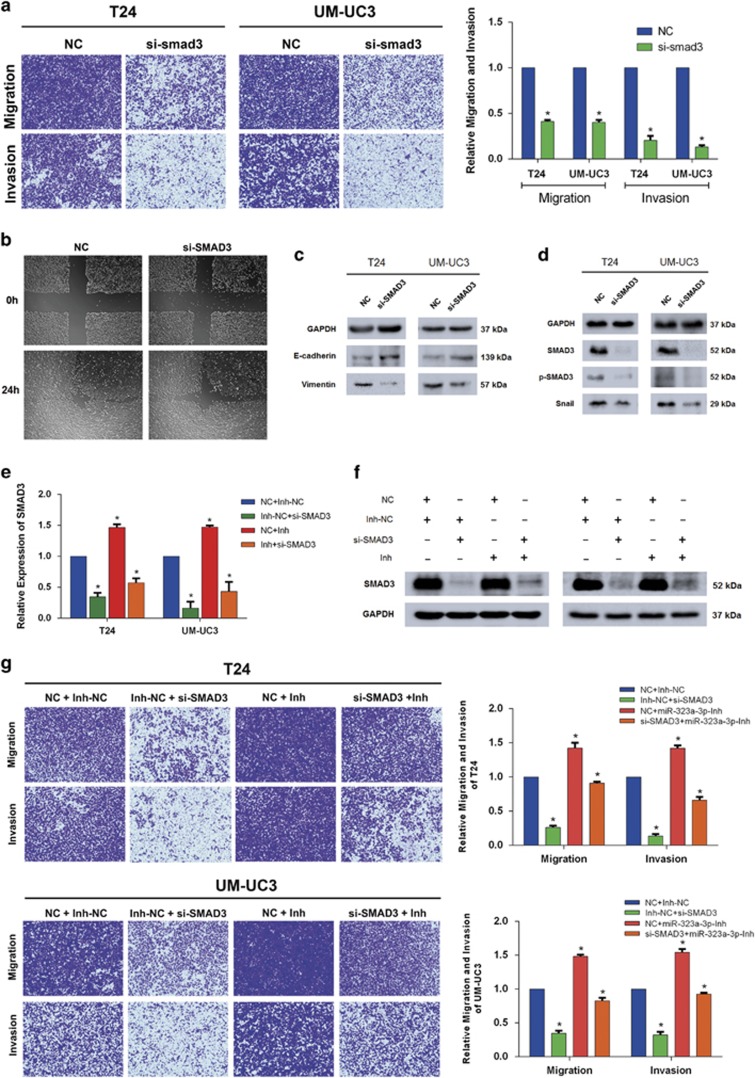
The knockdown of SMAD3 repressed the motility of T24 and UM-UC3 cell lines and inhibition of miR-323a-3p expression partially rescues si-SMAD3-induced suppression of cell motility. (**a**) Trans-well assay suggested the significant inhibition of motility, and the relative migration and invasion rates were calculated. (**b**) A wound healing assay revealed the consistent repression of the motility of T24 cell line. (**c** and **d**) Three SMAD3 siRNAs were merged together as siRNA-pool to transfect T24 and UM-UC3 cell lines and western blot assay revealed inhibition of EMT associated proteins and SNAIL protein. (**e**,**f**) Co-transfection of miR-323a-3p-Inh and si-SMAD3 attenuated the expression repressed by si-SMAD3 both at the mRNA and protein levels in T24 and UM-UC3 cell lines. (**g**) Trans-well assays consistently indicated co-transfection of miR-323a-3p and si-SMAD3 abrogated the motility of T24 and UM-UC3 cell lines. And the relative migration and invasion rate was calculated. Error bars represent the S.E. obtained from three independent experiments; *P<0.05. Photographs of the trans-well assay were obtained under × 10 objective

As a key transcription factor (TF), SMAD3 participated in the development of tumors especially the migration progression. And SMAD3 mutations and overexpression have been identified and observed in many cancers.^21^ However, the precise role of SMAD3 in regulating BCa was limited. We utilized si-SMAD3 to transfect T24 and UM-UC3 cell lines, and analyzed the migration and invasion capabilities by trans-well and wound healing assays, the results of which revealed that the migration and invasion was significantly inhibited by knockdown of SMAD3 (Figures 5a and b). To avoid off-target effects, we designed three different and effective siRNAs and the knockdown efficiencies at mRNA and protein levels were all tested by qRT-PCR and western blot analysis (Supplementary Figures S6A and B). Western blot analysis also demonstrated the corresponding changes of representative protein markers of EMT after knockdown of SMAD3 (Figure 5c). A further test of signal-associated protein suggested that knockdown of SMAD3 also reduced the expression of SNAIL (Figure 5d). Previous studies have elucidated that R-SMADs could interact with EMT-associated TFs such as SNAIL,SLUG, HMGA2 and ZEB1/2, resulting in the repression of E-cadherin and inducing EMT.^22^ Above evidence indicated that SNAIL as a downstream of SMAD3 consisted of a SMAD3/SNAIL/EMT circuit in the regulation of BCa.

To further confirm the direct interaction between SMAD3 and miR-323a-3p, we conducted two rescue experiments. In the first instance, we examined whether inhibition of miR-323a-3p could abrogate the inhibition of EMT progression induced by si-SMAD3. miR-323a-3p-inhibitor and si-SMAD3 were co-transfected into T24 and UM-UC3 cell lines, and we detected the significantly attenuated mRNA and protein level of SAMD3 (Figures 5e and f). And the migration and invasion tested by trans-well and wound healing assay showed a partial abrogation induced by miR-323a-3p-Inh (Figure 5g and Supplementary Figure 6C). Secondly, another rescue experiment with overexpressed plasmid (p-SMAD3) was also conducted and confirmed the same results. Trans-well assay suggested that only overexpression of SMAD3 significantly induced the migration and invasion, and co-transfection with overexpressed plasmid (p-SMAD3) and miR-323a-3p reversed the migration and invasion repressed by miR-323a-3p in T24 and UM-UC3 cell lines (Supplementary Figures S7 A and B). The changes of protein level were presented consistently with the phenotype (Supplementary Figure S7C).

To summarize, we demonstrated that SAMD3 as an important protein targeted by miR-323a-3p was involved in the progression of BCa.

### A circuit mediated by miR-323a-3p was established to regulate EMT progression in BCa

**Figure 6:**
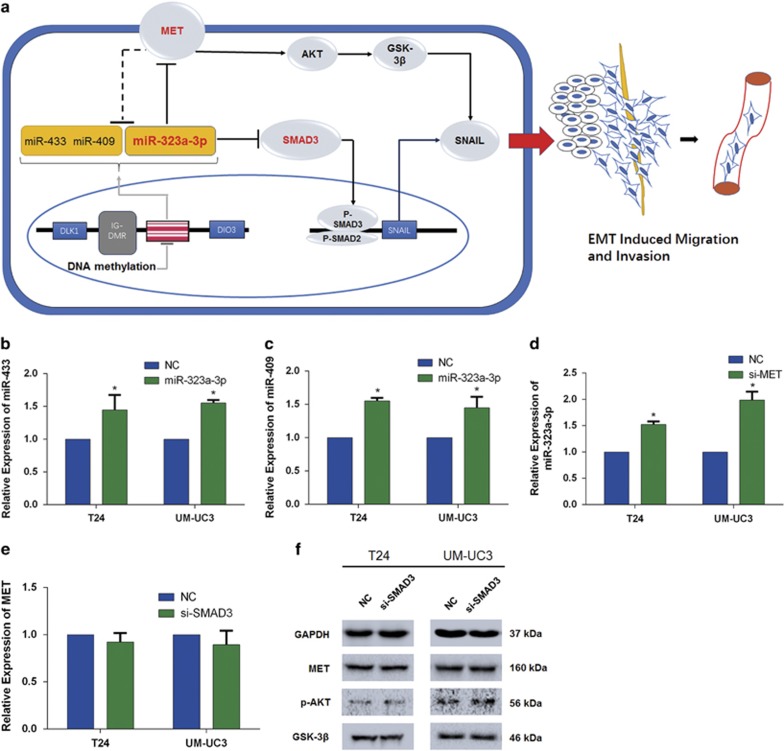
miR-323a-3p-mediated regulatory circuit in BCa. (**a**) The schematic diagram represented above includes miR-323a-3p/MET/SMAD3/SNAIL circuit, mutual regulation between three miRNAs (miR-323-3p, miR-433 and miR-409) and MET, and the dysregulation of miR-323a-3p induced by the methylation of DMR. (**b**–**e**) The mutual regulation between miR-323a-3p/miR-433/miR-409 and MET; however, SMAD3 may not reduce the level of miR-323a-3p. (**f**) Western blot analysis confirmed SMAD3 may not induce the expression of MET. Error bars represent the S.E. obtained from three independent experiments; *P<0.05

All of the above-mentioned results showed that the downregulation of miR-323a-3p induced by the methylation of the IG-DMR region in the upstream targeted both MET and SMAD3, which ultimately regulated SNAIL to repress EMT progression via two different pathways. Analyzing above results, we concluded that a regulatory circuit existed in BCa (Figure 6a). Previous studies have demonstrated the mutual regulation of MET, miR-409 and miR-433,^20, 23^ and thus we performed qRT-PCR to examine whether miR-323a-3p and MET are regulated in a similar manner. The results showed that the overexpression of miR-323a-3p induced the expression of miR-409 and miR-433 (Figures 6b and c). And the expression of miR-323a-3p was upregulated by knockdown of MET (Figure 6d); however, knockdown of SMAD3 could not influence the expression of miR-322a-3p (Figure 6e). Xianghong Zhang *et al.* reported that both Sp1 and SMADs (SMAD2, SMAD3, SMAD4) induced the expression of MET by binding to MET promotor (SMAD binding element, SBE) in renal epithelial cells.^24^ Recent studies found MET promoter contained a putative binding site for SMADs, and this binding activity was constitutively upregulated in systemic sclerosis fibroblasts as well as in normal fibroblasts treated with exogenous TGF-*β*1.^25^ Whether SMADs have the same effect on MET in tumors, especially BCa, no studies have elucidated. Therefore, we knocked down SMAD3; however, no significant reduction of MET expression was found, and the associated proteins of MET-induced signal were also not altered (Figure 6f). Collectively, SMAD3 did not bind to the promoter of MET to induce the expression of SNAIL, but rather, it regulated SNAIL in a direct way, which indicated the complexity of tumor progression. In summary, a novel miR-323a-3p/MET/SMAD3/SNAIL circuit and mutual regulation between miR-323a-3p/miR-433/mir-409 and MET were established to regulate EMT progression in BCa.

## Discussion

BCa is one of the most common cancers around the world. A surging number of patients are diagnosed and dead from BCa. Although the incidence rates are consistently higher in men than women, stabilizing or declining rates in men but some increasing trends were seen for women in many countries. Besides, deaths of smoking-related BCa are increasing among women in developed countries.^1, 2, 3, 4, 5^ The high incidence, mortality rates of BCa has severely endangered our health, it is urgent for us to investigate the specific mechanisms for a better treatment.

Emerging evidence has shown that miRNAs play a significant role in the development of various tumors in epigenetic way. miR-323a-3p belonging to the DLK1-DIO3 genomic imprinted miRNA cluster is located at chromosomal region 14q32.31, and functions as a tumor suppressor in several different types of human tumors such as prostate cancer and pancreatic ductal adenocarcinoma.^25, 26, 27^ However, its specific role in BCa has not been elucidated so far. In this study, we demonstrated miR-323a-3p as a tumor suppressor was downregulated in both BCa cell lines and tissues. And gain-of-function experiments revealed a significant inhibition of migration and invasion by regulating a MET/SMAD3/SNAIL circuit. All the findings highlighted the tumor suppressor role of miR-323a-3p in BCa.

High methylation status of CpG islands in promoters that is common in numerous tumors contributes to the silence of tumor suppressor genes.^28^ miR-323a-3p is a member of DLK1-DIO3 genomic imprinted miRNA cluster, and increasing evidence reveals that some miRNAs in this cluster are silenced by the upstream IG-DMR and MEG3-DMR.^17, 18, 29^ Therefore, we performed BSP sequencing, and the results revealed the high methylation level of IG-DMR in BCa cell line (T24, UM-UC3, 5637). At the same time, the treatment of above three cell lines with 5-aza-CdR obviously induced the expression of miR-323a-3p. Collectively, these epigenetic modifications are involved in the regulation of miR-323a-3p.

The EMT progression is executed in response to multiple signaling factors that induce the expression of specific TFs (e.g. SNAIL, ZEB, TWIST, and others) that are involved in cancer metastasis.^30^ Many EMT-associated inducers are known, but only a few pathways participated in the EMT progression. The first category is fibroblast growth factors and hepatocyte growth factor (HGF) which induce EMT via triggering receptor tyrosine kinases (RTKs). The second category is TGF-*β* family proteins that act through SMAD TFs. The third category is Wnts acting through *β*-catenin and TCF/LEF TFs. Others include Hedgehog proteins that activate Gli proteins. All the inducers cooperate with signaling pathways that coordinately direct changes to promote EMT.^31^ In terms of EMT in BCa, similar EMT-associated inducers (snail, slug, twist, zeb1,and zeb2) but different upstream factors trigger the EMT progression.^32^ GSK-3*β*/ZEB1 pathway activated by the upstream integrin-linked kinase significantly induce EMT in BCa.^33^ Conversely, forced expression of RASAL2 (RAS GTPase-activating protein) obviously inhibited BCa stemness and EMT.^34^ Non-coding RNAs also played great role in BCa EMT progression, multi-targets effects mediated by stable expression of miR-200 family in mesenchymal UMUC3 cells could upregulate E-cadherin levels, meanwhile, downregulate expression of ZEB1, ZEB2, ERRFI-1 to inhibit migration.^35^ Moreover, long-non-coding RNA metastasis-associated lung adenocarcinoma transcript 1 (malat1) is also associated with TGF-*β*-induced EMT of BCa in the regulation of SUZ12.^36^ In this study, we found that both MET and SMAD3 were targeted by miR-323a-3p to repress EMT progression. As an RTK, MET is frequently overexpressed in numerous human cancers and promotes tumor growth, invasion and dissemination.^37^ Recently, emerging evidence indicated that inhibition of MET signaling is regarded as a promising approach for cancer therapy.^38, 39^ Besides, multitudes of clinical trials of MET-targeted tharapy (HGF/MET pathway antagonists and small synthetic MET kinase inhibitors) were initiated and showed the satisfying and promising results in terms of antitumor efficacy and improvement of clinical outcomes with few adverse effects in various cancers (non-small-cell lung cancer, kidney, pancreatic cancer and pediatric glioblastoma).^40, 41, 42^ When referring to MET in BCa, some studies have remaindered its importance in the tumor progression. A study indicated that urinary soluble MET levels in patients with BCa are higher than those in patients without BCa and associated with disease progression, which implied MET as a potential diagnosis biomarker.^43^ Besides, Hass *et al.*^44^ has found that the abundance of MET expression and intracellular signaling is involved in the carcinogenesis of BCa. Previous studies in our research team have demonstrated that MET targeted by miR-433 and miR-409 significantly suppressed the EMT of BCa. In this study, miR-323a-3p also blocked the migration and invasion of BCa cell lines via targeting MET to regulate AKT/GSK-3*β*/SNAIL signaling, and rescue experiments further confirmed the direct interaction. The other target, SMAD3, as a TF and a key member of TGF-*β* signaling family, cooperates with other SMADs to activate the expression of downstream genes. Increasing studies have demonstrated that SMAD signaling is essential for TGF-*β*-induced EMT, and Smad3/Smad4 enhances the induction of EMT by activated TGF-*β* receptors.^45^ To conclude, surging lines of evidence indicated SMAD3 played a great role in tumor progression; however, compared to MET, SMAD3-targeted therapy was rarely researched, maybe it needs time to explore the more profound mechanisms in cancers. Genome-wide expression profile of BCa suggested that high expression of SMAD3 indicates a poor overall survival and is significantly associated with increase in tumor invasive depth.^46^ Dephosphorylate p-Smad2/3 induced by PPM1A has been confirmed to inhibit the EMT progression of BCa.^47^ All above indicates that SMAD3 as an important regulator may be involved in the EMT progression of BCa. However, the precise mechanism of how SMAD3 influences downstream to regulate EMT in BCa remained elusive. In this study, we detected that miR-323a-3p targeted SMAD3 to reduce EMT progression, and that the knockdown of SMAD3 with siRNAs obtained the consistent results with overexpressed miR-323a-3p. And surprisingly SNAIL was also downstream of SMAD3, which indicated that miR-323a-3p repressed EMT partially depending on SMAD3/SNAIL signaling. Previous studies have found that SMAD3 could bind to the SBE in the promotor of MET to induce the expression of MET in epithelial cells and also triggers systemic sclerosis fibroblasts.^24, 25^ To further explore whether SMAD3 could regulate MET in BCa in the same manner, western blot and qRT-PCR analysis were performed but the results suggested no significant reduction of MET in SMAD3 knockdown BCa cells, which indicated that SMAD3 could not regulate MET directly binding to its promotor. In conclusion, the repression of EMT induced by miR-323a-3p is mediated by a miR-323a-3p/MET/SMAD3/SNAIL circuit, and reduction of SNAIL is the last confocal protein of two pathways that induce the loss of E-cadherin, subsequently the EMT progression.

Previous studies have shown that the overexpression of MET also reversely reduce the expression of miR-433 and miR-409 indirectly and these two miRNAs could regulate mutually.^20, 23^ In this study, we also found that MET could regulate the level of miR-323a-3p which is the fourth miRNA that has been discovered to be regulated by MET in BCa up to now. Moreover, miR-323a-3p similarly induces the expression of both miR-433 and miR-409. All these results are consistent with the findings of a previous study that MET could regulate the 14q32.2 miRNA cluster. However, SMAD3 was not possessed of this capability to regulate the expression of miR-323a-3p. Collectively, the regulation of the miRNAs cluster in DLK1-DIO3 genomic region was not only dependent on the methylation of IG-DMR or MEG3-DMR, but was also strongly associated with its target (MET); furthermore, the mutual induction of miRNAs in this cluster was also of great significance, because it indicates that an extensive regulatory network in this region is involved in the progression of BCa. And all of the results discussed above acquire a further profound investigation.

In summary, we found that miR-323a-3p functions as suppressor to inhibit EMT progression by targeting MET/SMAD3/SNAIL circuit. The specific points are listed as follows: (i) miR-323a-3p is frequently downregulated in BCa due to altered DNA methylation of IG-DMR, which indicates a poor overall survival rate of BCa; (ii) miR-323a-3p functions as a tumor suppressor to inhibit the EMT progression in BCa cells; (iii) MET and SMAD3 are identified as downstream target genes of miR-323a-3p; (iv) miR-323a-3p inhibits EMT in BCa cells by regulating MET/SMAD3/SNAIL circuit and (v) a mutual regulation may exist between MET and miR-323a-3p/miR-433/miR-409. Our study has revealed the significant role of miR-323a-3p in BCa progression and we expect that our findings on the miR-323a-3p/MET/SMAD3/SNAIL circuit will provide useful references or a basic support for the more effective and promising therapies against BCa in the future.

## Materials and Methods

### Cell lines and cell culture

T24, UM-UC3 and 5637 human BCa cell lines and the normal bladder epithelial cell line SV-HUC-1 were purchased from the Cell Bank of Type Culture Collection of the Chinese Academy of Sciences (Shanghai, China). All cell lines were verified by short tandem repeat DNA profiling analysis. T24 and 5637 cell lines were cultured in RPMI 1640 medium, UM-UC3 cell line was cultured in MEM medium, and SV-HUC-1 cell line was cultured in F12K medium at 37 °C under a humidified atmosphere of 5% CO_2_. Additional 10% heat-inactivated fetal bovine serum (FBS) was added to the above media.

### Human clinical samples

Paired BCa tissues and adjacent non-tumorous bladder mucosal tissues were obtained from patients undergoing radical cystectomy. The samples were collected between January 2011 and October 2013 at the First Affiliated Hospital of Zhejiang University, after informed consent and approval of Ethics Committee. Tissue samples were snap frozen in liquid nitrogen until RNA extraction.

### Reagents and transfection

RNA duplexes were chemically synthesized by GenePharma Company (Shanghai, China). All of the corresponding sequences are listed as follows: miR-323a-3p mimic (sense): 5′-CACAUUACACGGUCGACCUCU-3′, NC (sense) 5′-ACTACTGAGTGACAGTAGA-3′, si-MET-1 (sense): 5′-GGAAAGAACCUCUCAACAUdTdT-3′, si-MET-2 (sense): 5′-GCAACAGCUGAAUCUGCAAdTdT-3′, si-MET-3 (sense): GGUGUUGUCUCAAUAUCAAdTdT-3′, si-SMAD3-1 (sense): 5′-GCGUGAAUCCCUACCACUAdTdT-3′, si-SMAD3-1 (sense): 5′-GCCAUCCAUGACUGUGGAUdTdT-3′, si-SMAD3-3 (sense): 5′- CCGCAUGAGCUUCGUCAAAdTdT-3′. In order to achieve the better knockdown efficiency and fewer off-targets effects, three siRNAs were merged together as an siRNA-pool to co-transfect BCa cell lines in all the interference experiments. And above oligonucleotide transfections were performed using Lipofectamine 2000 reagents (Invitrogen, Carlsbad, CA, USA) in accordance with the manufacturer’s protocol. FuGENE HD Transfection Reagent (Promega, Madison, WI, USA) was applied for all plasmids transfection according to the manufacturer’s protocol.

### RNA isolation and qRT-PCR

RNA was extracted from cell lines with RNAiso plus (TaKaRa, Kusatsu, Japan) and subsequently transcribed into cDNA with PrimeScript RT reagent Kit and a One Step PrimeScript miRNA cDNA Synthesis Kit. The expression levels of miRNA and mRNA were detected by qRT-PCR. SYBR Premix Ex Taq (TaKaRa, Kusatsu, Japan) was used to quantify the transcribed cDNA with the ABI 7500 fast real-time PCR System (Applied Biosystems, Carlsbad, CA, USA). Small nuclear RNA U6 and GAPDH mRNA were used as endogenous references to calculate the relative expression of associated genes with 2^-ΔΔCt^ (delta-delta-Ct algorithm) method. All primers used were listed in Supplementary Table S2.

### Western blot analysis

All proteins were extracted from transfected cell lines with RIPA lysis buffer, and relative concentrations were quantified with a BCA Protein Assay kit. Subsequently, the extracted proteins were loaded onto 10% SDS-polyacrylamide gels and fully electrophoresed. After full separation, the proteins were transferred to PVDF membranes with wet transfer method. The membrane was then incubated with the primary antibody (at 1:1000 dilution) at 4 °C overnight after blocking with 5% fat-free milk for 1 h. The membrane was washed with TBS-Tween buffer three times for 30 min, and was then immersed in secondary antibody (diluted with diluent as 1:5000 ratio) for 1 h at room temperature. After three washes in TBS-Tween buffer, the protein level was detected with enhanced chemi-luminescence system (Pierce Biotechnology Inc., Rockford, IL, USA). The primary antibodies used are listed as follows: anti-GAPDH, anti-GSK-3*β*, anti-p-GSK-3*β*, anti-SMAD3, anti-E-cadherin, anti-N-cadherin, anti-Vimentin, anti-SNAIL (Cell Signaling Technology, Beverly, MA, USA), anti-MET, anti-p-AKT (Epitomics, Burlingame, CA, USA). The fold changes of band intensity in all western blot experiments were quantified with ImageJ (intensity-measured tool) and presented in supplementary figure S8.

### Dual-luciferase reporter assay

We designed and ordered the oligonucleotide pairs containing the desired miR-323a-3p target region or mutant target region from Sangon, Shanghai, China. After the annealing step, these double-stranded segments were inserted into the pmirGLO Dual-Luciferase miRNA Target Expression Vector (Promega), between the *Sac*I and *Sal*I sites. DNA sequencing was employed to verify the insertions. T24 and UM-UC3 cell lines were seeded in 24-well plates and were co-transfected with 50 nM miR-323a-3p or NC and 100 ng reporter pmirGLO. The relative luciferase activity was measured by the Dual-Luciferase Reporter Assay System (Promega) 48 h after transfection.

### Trans-well assay

A trans-well assay was used to evaluate cell migration and invasion. The matrix gel mimicking the *in vivo* matrix around tumor cells was plated at the bottom of the trans-well chamber, and approximately 5 × 10^4^ T24 cells and 8 × 10^4^ UM-UC3 cells (transfected with NC and mimics or siRNAs) suspended in 0.2 ml serum-free medium were placed onto the surface layer of the matrix gel. The entire chamber was placed into a 24-well plate, and 600 *μ*l RPMI-1640 medium supplemented with 10% FBS was added to the space between the chamber and the well. After incubation for 24 h at 37 °C, we detected the invasion rate using methanol and 0.1% crystal violet treatment. However, the migration rate was determined without the matrix gel step. Photographs were obtained under phase-contrast microscopy (Olympus, Tokyo, Japan) with a × 10 objective.

### Wound healing assay

When transfected cells were grown to 100% confluence in six-well plates, a micropipette tip was used to make a cross wound and wound healing was observed after 24 h culture. Photographs were obtained under phase-contrast microscopy (Olympus)

### 5-aza-CdR treatment and DNA methylation analysis

T24 and UM-UC3 cell lines were treated with 5 *μ*M 5-aza-CdR (Sigma, St Louis, MO, USA) for 4 days. qRT-PCR was performed to test the expression of miR-323a-3p. Following the bisulfite conversion, the CpG islands of miR-323a-3p in IG-DMR were amplified by PCR with primers 5′-TTAAGAGTTTGTGGATTTGTGAGAA-3′ (forward) and 5′- TCACAATAAACTACACTACTAAAAACTACA-3′ (reverse). Subsequently the PCR products were cloned into the pUC18 T-vector. After bacterial amplification, all the clones were sent to DNA sequencing (Sangon).

### Immuno-histochemical (IHC) staining

TMAs were purchased from Xinchao Biotech, Shanghai, China, which contained 46 cases with paired tumor and non-tumor tissues and 13 cases without corresponding non-tumor tissues. All paraffin tissue sections were dewaxed and rehydrated. Antigen retrieval was performed by heating the slides in sodium citrate buffer (10 mM, pH 6.0). After blocking with bovine serum albumin (Sango Biotech, Shanghai, China), the slides were incubated with anti-SMAD3 (Cell Signaling Technology) overnight at 4 °C. The slides were then incubated with a secondary antibody of goat anti-rabbit HRP conjugate (Cell Signaling Technology) for 1 h at room temperature. A DAB solution was used for brown color development. Both the intensity and proportion of positive cells were considered for the semi-quantification of the strength of positivity.

### Chromogenic *in situ* hybridization (CISH)

A 5′-DIG and 3′-DIG-labeled, locked nucleic acid-incorporated miRNA probe (miRCURY LNATM Detection probe, Exiqon, Woburn, MA, USA) was applied for the detection of miR-323a-3p in BCa TMAs. The probe sequence of hsa-mir-323a-3p was designed as follows: 5′-AGAGGTCGAC CGTGTAATGTG-3′. The specific manipulations were performed as previously described.^48^ Both the intensity and proportion of positive cells were considered for the semi-quantification of the strength of positivity.

### Statistical analysis

The data were expressed as the means±S.D. Differences between groups were estimated using the *χ*^2^-test or Student’s *t*-test. Overall survival rates were calculated according to the Kaplan–Meier method with log-rank test. A Cox regression analysis (proportional hazards model) was performed for the multivariate analyses of prognostic factors. All analyses were conducted using SPSS 16.0 software (IBM, Armonk, NY, USA), and significance was defined as a two-tailed value of *P*<0.05.
