# Supplementary material for: MET/SMAD3/SNAIL circuit mediated by miR-323a-3p is involved in regulating epithelial–mesenchymal transition progression in bladder cancer
Source: Cell Death Dis. 2017 Aug 24;8(8):e3010–. doi: 10.1038/cddis.2017.331 (PMC5596538; doi:10.1038/cddis.2017.331)
Supplement: Supplementary Table 1 [file cddis2017331x1.docx]

| **Table S1. Clinical data of the patients.** |
| --- |

| Patient no. | Sex | Age | TNM stage | Histological grade |
| --- | --- | --- | --- | --- |
| 1 | Male | 86 | T1N0M0 | III |
| 2 | Male | 72 | T3N0M0 | III |
| 3 | Male | 53 | T1N0M0 | III |
| 4 | Male | 62 | T2N0M0 | III |
| 5 | Male | 56 | T2N0M0 | III |
| 6 | Female | 74 | T2N0M0 | III |
| 7 | Male | 55 | T1N0M0 | II |
| 8 | Female | 76 | T3N0M0 | III |
| 9 | Male | 65 | T2N0M0 | II |
