# Supplementary material for: MET/SMAD3/SNAIL circuit mediated by miR-323a-3p is involved in regulating epithelial–mesenchymal transition progression in bladder cancer
Source: Cell Death Dis. 2017 Aug 24;8(8):e3010–. doi: 10.1038/cddis.2017.331 (PMC5596538; doi:10.1038/cddis.2017.331)
Supplement: Supplementary Table 2 [file cddis2017331x2.docx]

**Table S2: The primers used in this study.**

| Name^a^ | Sequence(5′->3′) |
| --- | --- |
| c-Met F | TGTCCCGAGAATGGTCATAA |
| c-Met R | AGGGAAGGAGTGGTACAACA |
| SMAD2 F | CGTCCATCTTGCCATTCACG |
| SMAD2 R | CTCAAGCTCATCTAATCGTCCTG |
| SMAD3 F | TGGACGCAGGTTCTCCAAAC |
| SMAD3 R | CCGGCTCGCAGTAGGTAAC |
| CREB1 F | ATTCACAGGAGTCAGTGGATAGT |
| CREB1 R | CACCGTTACAGTGGTGATGG |
| MITF F | TTATAGTACCTTCTCTTTGCCAGTCC |
| MITF R | GTTTATTTGCTAAAGTGGTAGAAAGGTACT |
| AKT3 F | GGAGTCATCATGAGCGATGTT |
| AKT3 R | AAGGAAGTATCTTGGCCTCCA |
| FGF2 F | ATGGCTCCCTTAGCCGAAGT |
| FGF2 R | AGGAAATGCGAACCCACCTG |
| CCND1 F | GCTGCGAAGTGGAAACCATC |
| CCND1 R | CCTCCTTCTGCACACATTTGAA |
| STAT3 F | CAGCAGCTTGACACACGGTA |
| STAT3 R | AAACACCAAAGTGGCATGTGA |
| CUL4a F | ACCTCGCACAGATGTACCAG |
| CUL4a R | AGGTTGACGAACCGCTCATTC |
| GAPDH F | AAGGTGAAGGTCGGAGTCA |
| GAPDH R | GGAAGATGGTGATGGGATTT |
| miR-323a-3p | CACATTACACGGTCGACCTCT |
| miR-433 | ATCATGATGGGCTCCTCGGTGT |
| miR-409-3p | GAATGTTGCTCGGTGAACCCCT |
| U6 F | TGCGGGTGCTCGCTTCGGCAGC |
| MET-utr-wt F | CATGTTTATAAATGAACAGGATGTAATGTACATAGATGACATTAAGAAAAG |
| MET-utr-wt R | TCGACTTTTCTTAATGTCATCTATGTACATTACATCCTGTTCATTTATAAACATGAGCT |
| MET-utr -mut F | CATGTTTATAAATGAACAGGATCATTACAACATAGATGACATTAAGAAAAG |
| MET-utr-mut R | TCGACTTTTCTTAATGTCATCTATGTTGTAATGATCCTGTTCATTTATAAACATGAGCT |
| SMAD2-utr-wt F | CCAGTAGTAGTTATGTGTACAGGTAATGTATCATGATCCAGTATCACAGTAG |
| SMAD2-utr-wt R | TCGACTACTGTGATACTGGATCATGATACATTACCTGTACACATAACTACTACTGGAGCT |
| SMAD2-utr-mut F | CCAGTAGTAGTTATGTGTACAGCATTACAATCATGATCCAGTATCACAGTAG |
| SMAD2-utr-mut R | TCGACTACTGTGATACTGGATCATGATTGTAATGCTGTACACATAACTACTACTGGAGCT |
| SMAD3-utr-wt F | CTTTAATGCAGAAGTAATGTATACTCTAGTATTCTGGTGTTTTTATATTTAG |
| SMAD3-utr-wt R | TCGACTAAATATAAAAACACCAGAATACTAGAGTATACATTACTTCTGCATTAAAGAGCT |
| SMAD3-utr-mut F | CTTTAATGCAGAACATTACAATACTCTAGTATTCTGGTGTTTTTATATTTAG |
| SMAD3-utr-mut R | TCGACTAAATATAAAAACACCAGAATACTAGAGTATTGTAATGTTCTGCATTAAAGAGCT |
| STAT3-utr-wt F | CTAGACTTATTTTCCTTTGTAATGTATTGGCCTTTTAGTGAGTAAG |
| STAT3-utr-wt R | TCGACTTACTCACTAAAAGGCCAATACATTACAAAGGAAAATAAGTCTAGAGCT |
| STAT3-utr-mut F | CTAGACTTATTTTCCTTTCATTACAATTGGCCTTTTAGTGAGTAAG |
| STAT3-utr-mut R | TCGACTTACTCACTAAAAGGCCAATTGTAATGAAAGGAAAATAAGTCTAGAGCT |

a: F, forward primer; R, reverse primer.
