# Supplementary material for: MET/SMAD3/SNAIL circuit mediated by miR-323a-3p is involved in regulating epithelial–mesenchymal transition progression in bladder cancer
Source: Cell Death Dis. 2017 Aug 24;8(8):e3010–. doi: 10.1038/cddis.2017.331 (PMC5596538; doi:10.1038/cddis.2017.331)
Supplement: Supplementary Table 3 [file cddis2017331x3.docx]

**Table S3****. Kaplan–Meier model analysis and multivariate Cox model analysis of overall survival of BCa.**

| **Variables** | **K-M analysis ^a^ P-value** | **Multivariable Cox analysis**  **HR^b^(95% CI) P-value** | |
| --- | --- | --- | --- |
| **Sex**  Female vs. male  **Age (years)**  >67 vs. ≤67  **Tumor grade**  High vs. low  **T stage**  T3-T4 vs. Tis-T2  **Lymph node metastasis** Positive vs. negative  **miR-323a-3p**  High vs. low  **SMAD3**  High vs. low | 0.769  0.799  0.752  0.119  0.024  0.003  0.702 | 1.015 (0.360-2.865)  1.010 (0.963-1.058)  1.458 (0.158-13.462)  1.303 (0.517-3.284)  2.961 (1.013-8.658)  0.262 (0.070-0.983)  0.978 (0.196-4.887) | 0.978  0.686  0.740  0.575  0.047  0.047  0.978 |

**^a^ K-M analysis:** Kaplan–Meier analysis (log-rank test)

^b^ **HR**: hazard ratio
