# Supplementary figures and images for: MET/SMAD3/SNAIL circuit mediated by miR-323a-3p is involved in regulating epithelial–mesenchymal transition progression in bladder cancer
Source: Cell Death Dis. 2017 Aug 24;8(8):e3010–. doi: 10.1038/cddis.2017.331 (PMC5596538; doi:10.1038/cddis.2017.331)

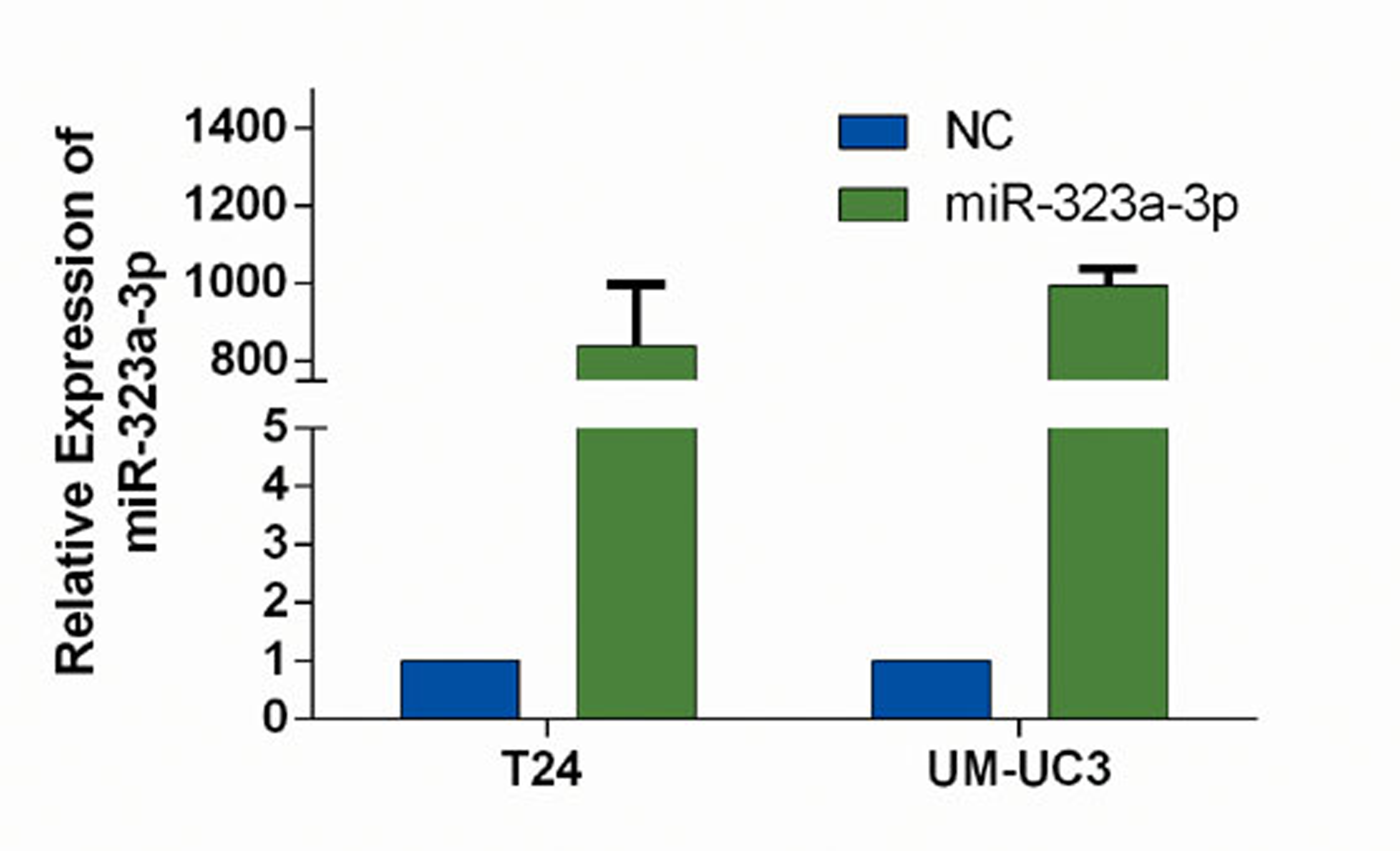

Supplement: Supplementary Figure 1 [file cddis2017331x4.tif]

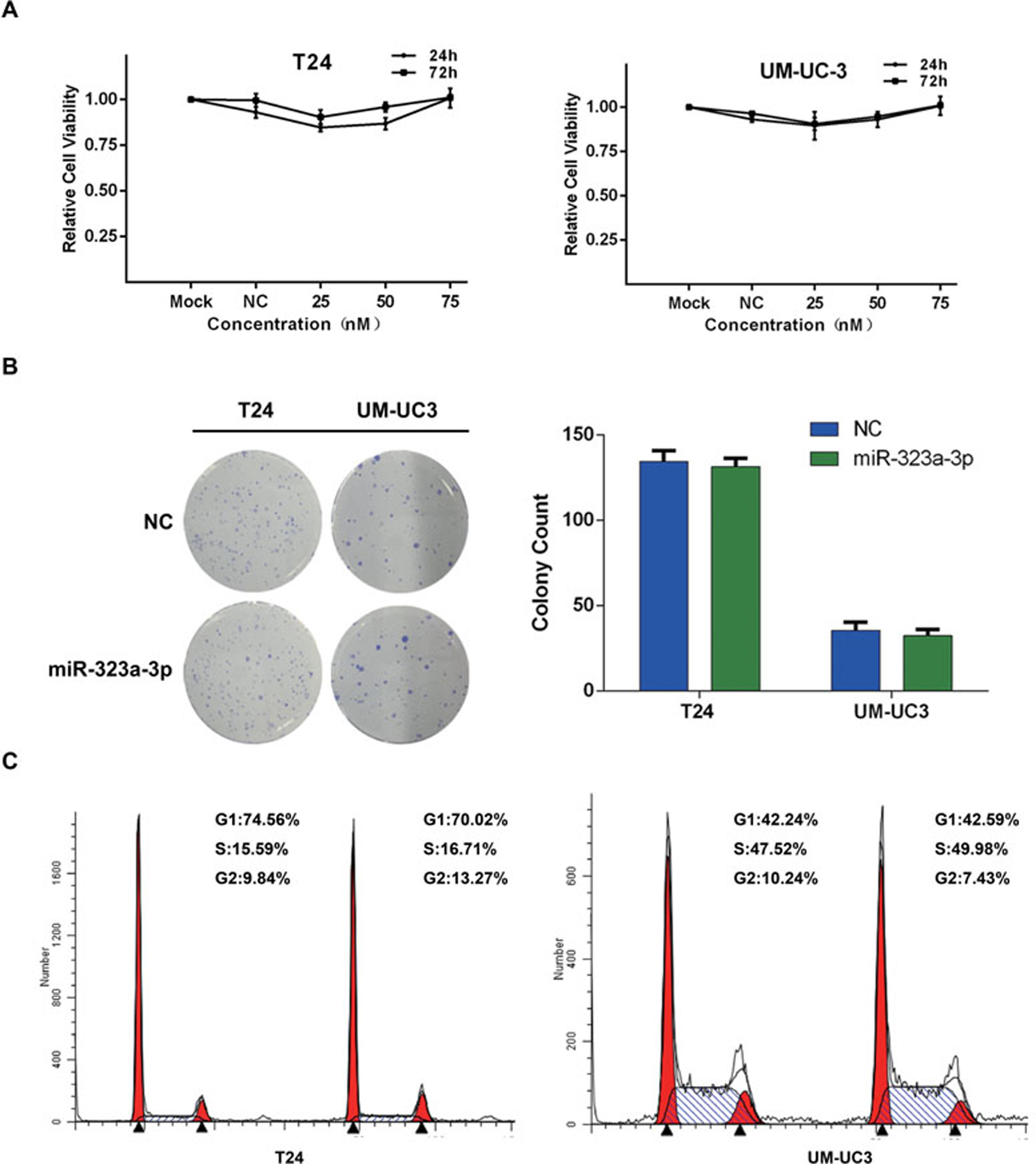

Supplement: Supplementary Figure 2 [file cddis2017331x5.tif]

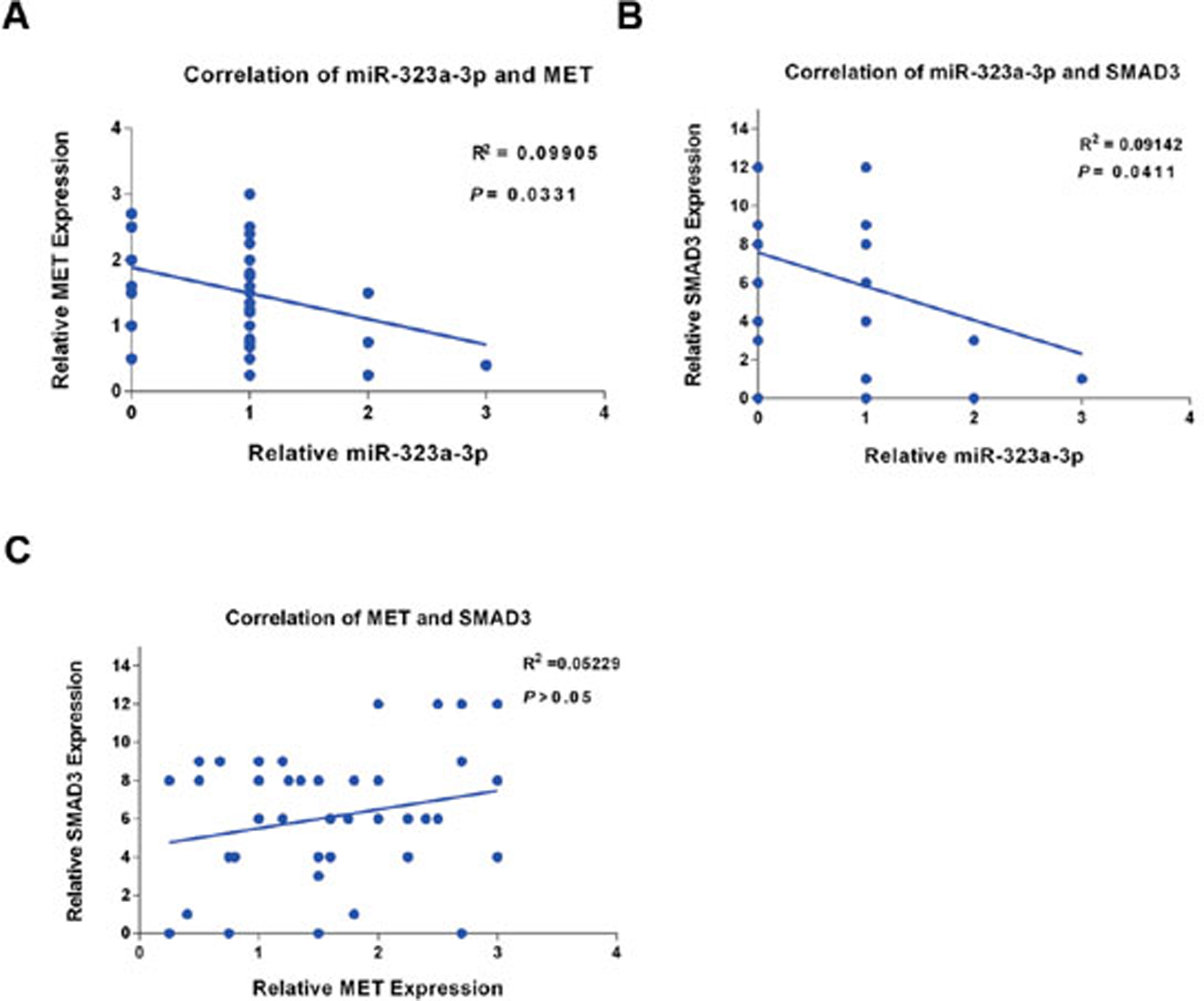

Supplement: Supplementary Figure 3 [file cddis2017331x6.tif]

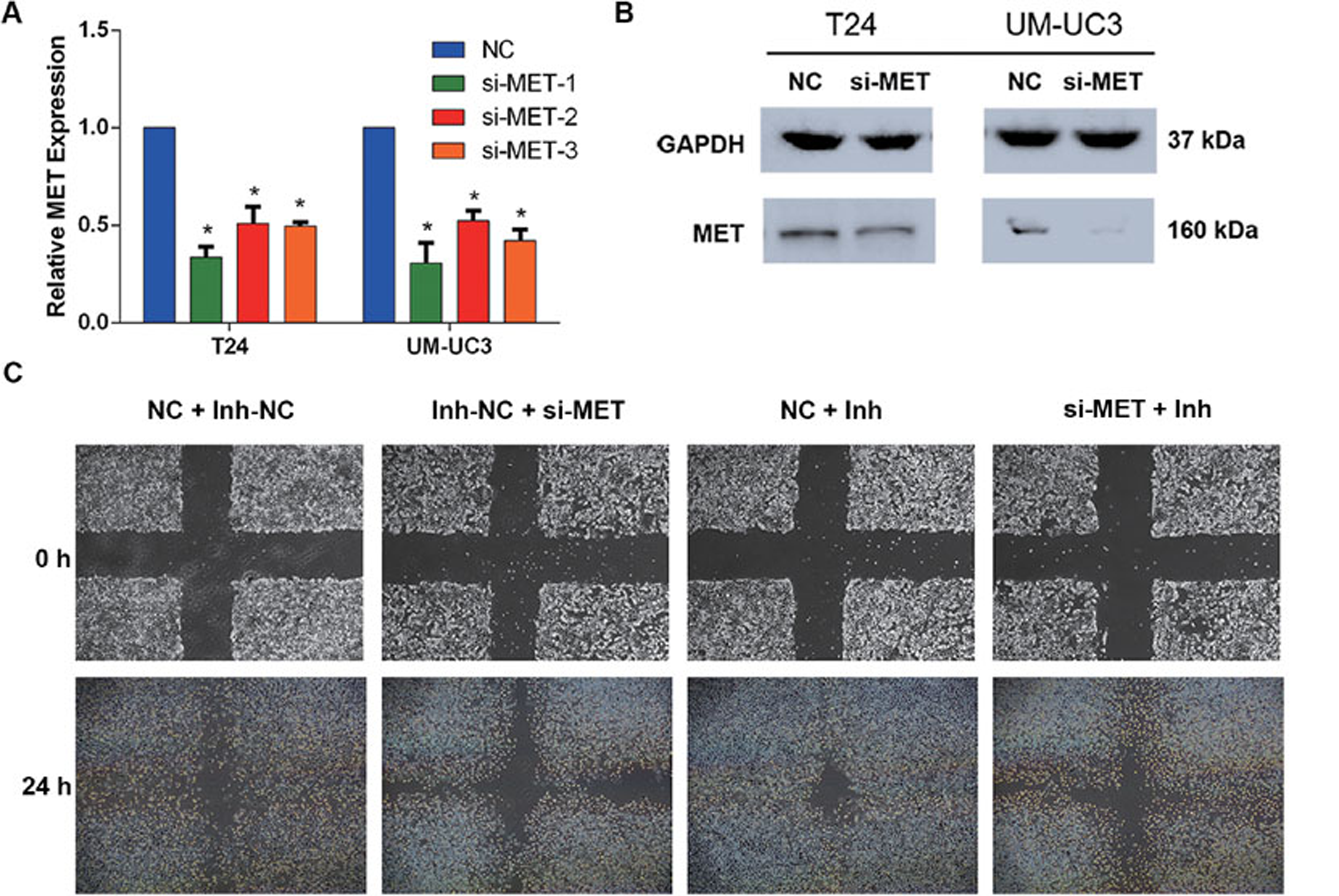

Supplement: Supplementary Figure 4 [file cddis2017331x7.tif]

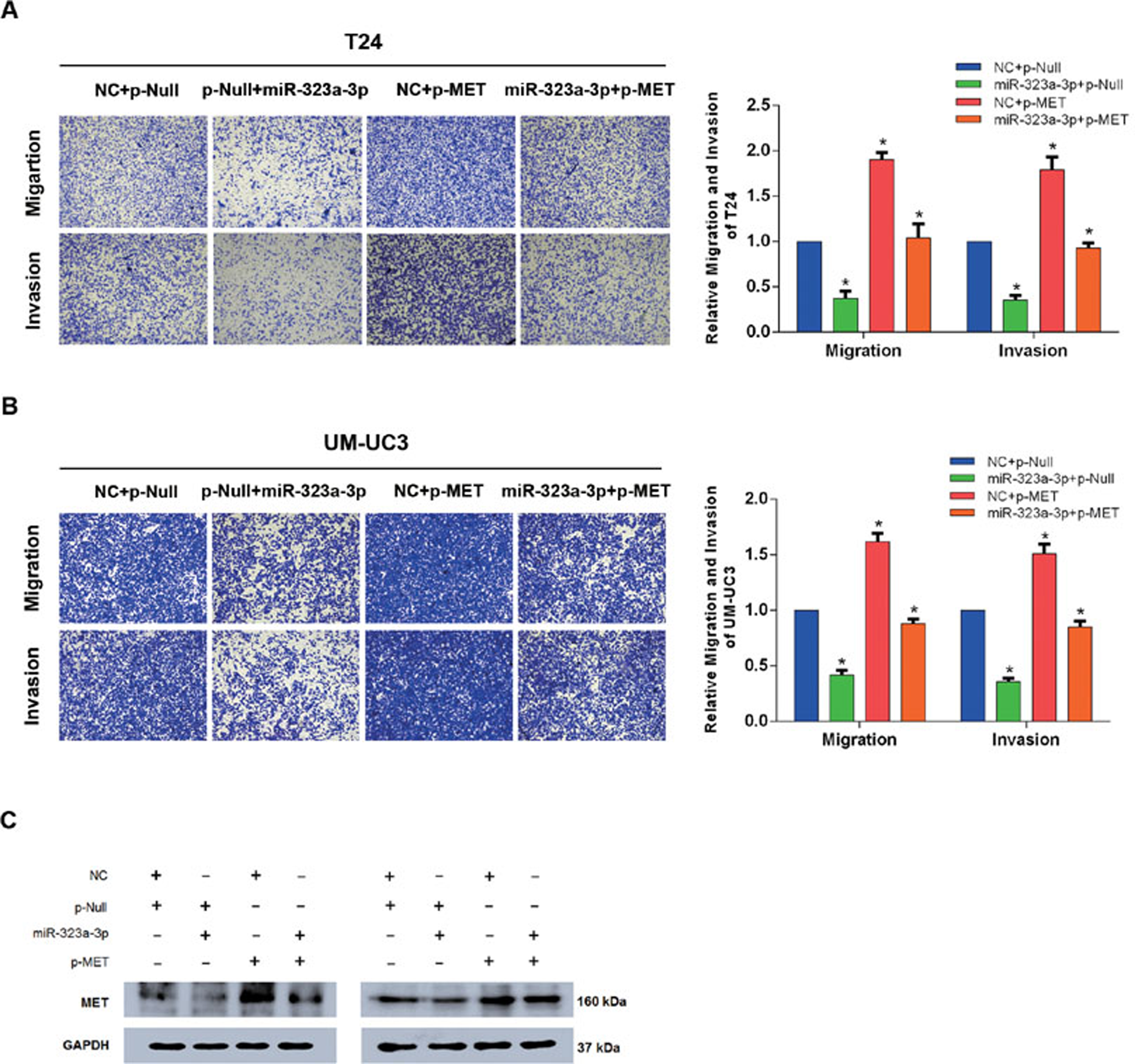

Supplement: Supplementary Figure 5 [file cddis2017331x8.tif]

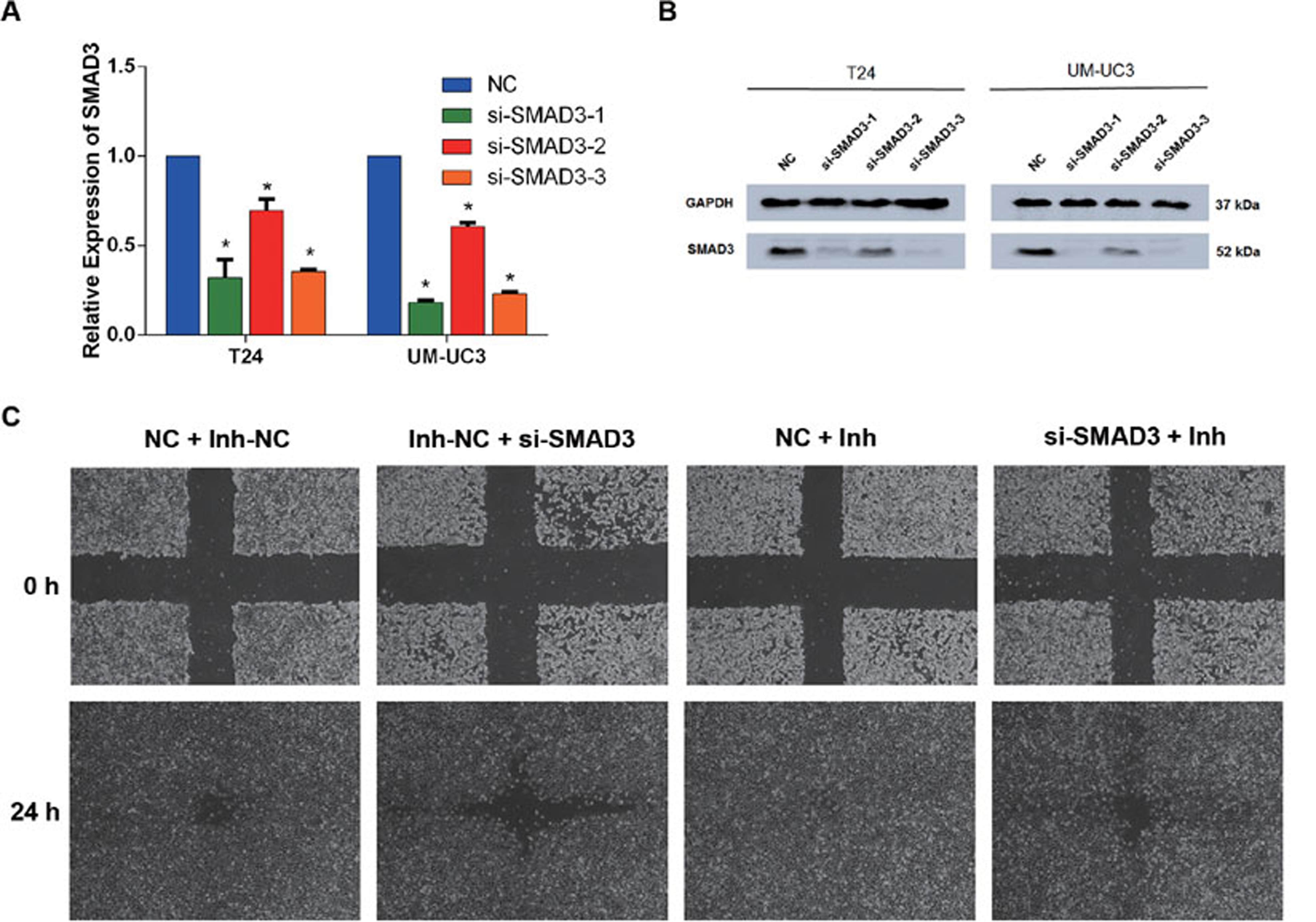

Supplement: Supplementary Figure 6 [file cddis2017331x9.tif]

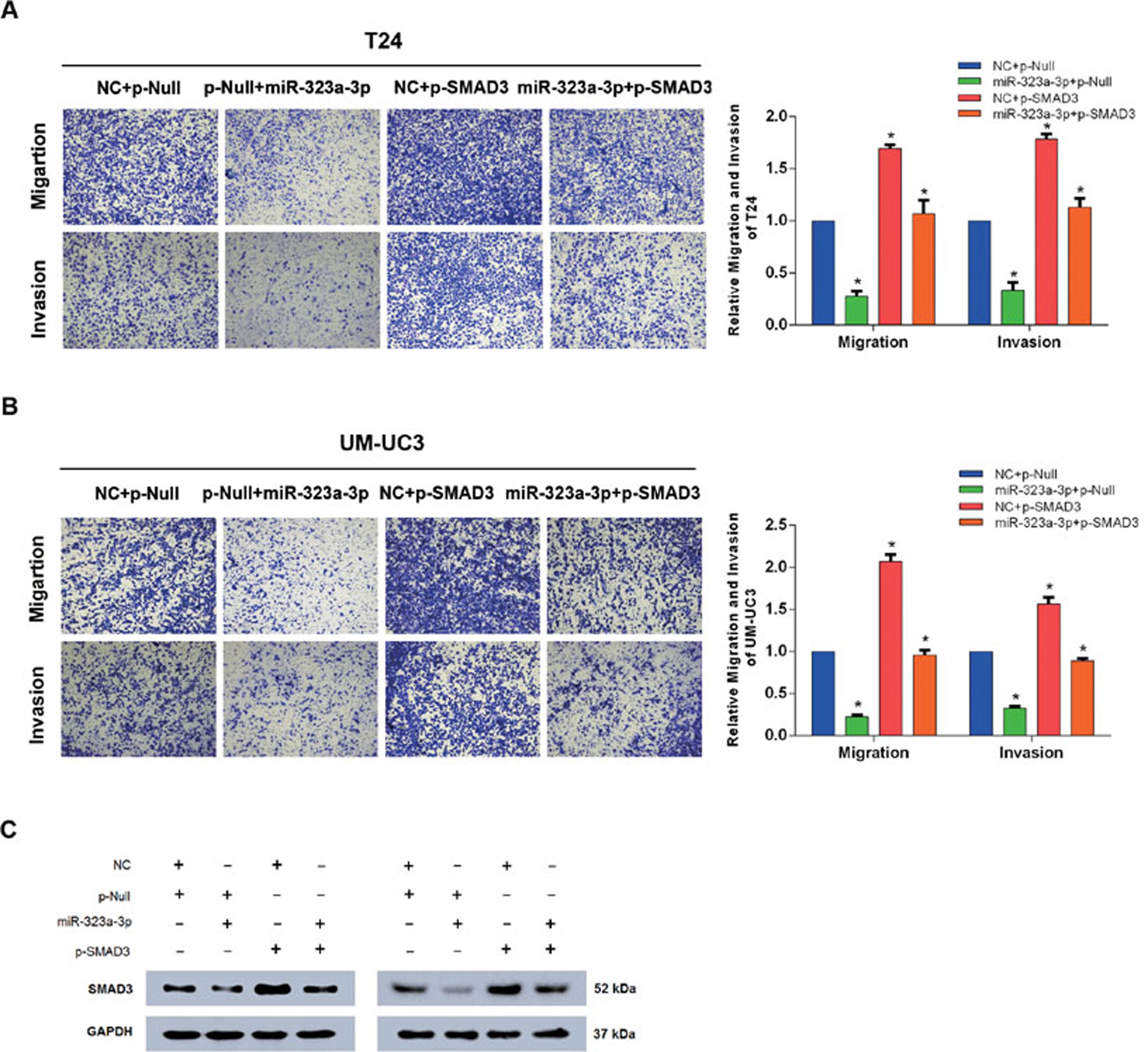

Supplement: Supplementary Figure 7 [file cddis2017331x10.tif]

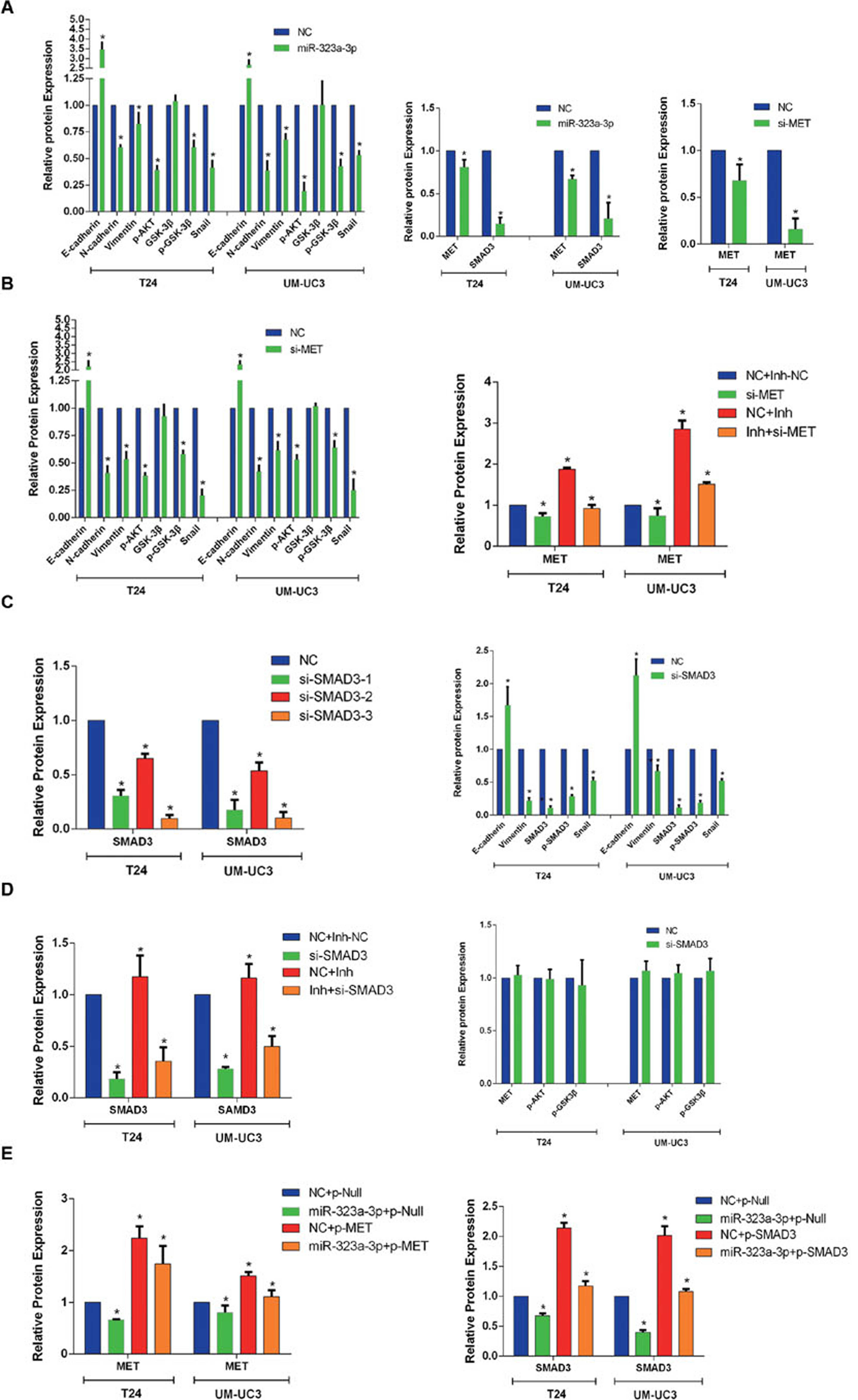

Supplement: Supplementary Figure 8 [file cddis2017331x11.tif]
