# Supplementary material for: MET/SMAD3/SNAIL circuit mediated by miR-323a-3p is involved in regulating epithelial–mesenchymal transition progression in bladder cancer
Source: Cell Death Dis. 2017 Aug 24;8(8):e3010–. doi: 10.1038/cddis.2017.331 (PMC5596538; doi:10.1038/cddis.2017.331)
Supplement: Supplementary Figure Legends [file cddis2017331x12.docx]

**Figure legends for Supplementary Figures 1-8**

Figure S1. The ectopic expression of miR-323a-3p is confirmed by qRT-PCR. Error bars represent the standard error obtained from three independent experiments; **P* < 0.05.

Figure S2. miR-323a-3p shows no significant inhibition on proliferation in two BCa cell lines. Error bars represent the standard error obtained from three independent experiments; **P* < 0.05.

Figure S3 The correlation with miR-323a-3p/MET/SMAD3 expression in TMAs. (A) The level of miR-323a-3p was significantly negatively correlated with MET. (B) The level of miR-323a-3p was significantly negatively correlated with SMAD3. (C) The level of MET and SMAD3 had a minor positive correlation tendency, however, no statistical significance.

Figure S4. The interference of si-MET is confirmed and the wound healing assay of rescue experiments is represented. (A) Three individual si-MET showed a significant inhibition of MET at mRNA level. (B) Three individual si-MET was merged as a RNA-pool to interference MET, and significant repression of MET was shown. (C) Wound haling confirmed the direct interaction between miR-323a-3p and MET with rescue experiments. Error bars represent the standard error obtained from three independent experiments; **P* < 0.05.

Figure S5. Overexpression of MET significantly reversed miR-323a-3p-repressed EMT phenotype. (A) The trans-well assay showed that overexpression of MET (p-MET) significantly reversed the miR-323a-3p-repressed migration and invasion in T24 cell line. (B) The trans-well assay showed that overexpression of MET (p-MET) significantly reversed the miR-323a-3p-repressed migration and invasion in UM-UC3 cell line. (C) The protein level changes of rescue experiments. Error bars represent the standard error obtained from three independent experiments; **P* < 0.05.

Figure S6. The interference of si-SMAD3 is confirmed and the wound healing assay of rescue experiments is represented. A and B. Three individual si-SMAD3 show a significant inhibition of SMAD3 at mRNA and protein levels. C. Wound healing confirmed the direct interaction between miR-323a-3p and SMAD3 with rescue experiments. Error bars represent the standard error obtained from three independent experiments; **P* < 0.05.

Figure S7. Overexpression of SMAD3 significantly reversed miR-323a-3p-repressed EMT phenotype. (A) The trans-well assay showed that overexpression of SMAD3 (p-SMAD3) significantly reversed the miR-323a-3p-repressed migration and invasion in T24 cell line. (B) The trans-well assay showed that overexpression of SMAD3 (p-SMAD3 ) significantly reversed the miR-323a-3p-repressed migration and invasion in UM-UC3 cell line. (C) The protein level changes of rescue experiments. Error bars represent the standard error obtained from three independent experiments; **P* < 0.05.

Figure S8. The fold changes of band intensity in all western blot experiments. (A) The fold changes of band intensity in Figure 2. (B) The fold changes of band intensity in Figure 3. (C) The fold changes of band intensity in Figure S4. (D) The fold changes of band intensity in Figure 4. (E) The fold changes of band intensity in Figure S6. (F,G) The fold changes of band intensity in Figure 5.(H) The fold changes of band intensity in Figure 6. (I) The fold changes of band intensity in Figure S5 and 7. Error bars represent the standard error obtained from three independent experiments; **P* < 0.05.
